# Promoting Adolescent Sexual and Reproductive Health in North America Using Free Mobile Apps: Environmental Scan

**DOI:** 10.2196/33826

**Published:** 2022-10-04

**Authors:** James Russell Andrew Benoit, Samantha Louie-Poon, Samar Kauser, Salima Meherali

**Affiliations:** 1 Faculty of Nursing University of Alberta Edmonton, AB Canada

**Keywords:** mHealth, mobile health, adolescent, sexual and reproductive health, environmental scan, mobile app, sexual health, reproductive health, health, sexual, reproductive, MARS, Mobile App Rating Scale, digital health, adolescents

## Abstract

**Background:**

Neglecting adolescents’ sexual and reproductive health (SRH) can affect multiple domains of development. Promoting healthy adolescent SRH is increasingly done using mobile phone apps. Providing SRH information via mobile phones can positively influence SRH outcomes including improving knowledge, reducing sexual risk behavior, and increasing the use of health services. A systematic approach to establishing and evaluating the quality of adolescent SRH mobile apps is urgently needed to rigorously evaluate whether they are a viable and effective strategy for reaching adolescents and improving adolescent SRH knowledge and behaviors in particular.

**Objective:**

This study aimed to conduct an environmental scan to produce an inventory of adolescent SRH–specific mobile apps with descriptions of their purpose, structure, operations, and quality of evidence.

**Methods:**

We used a literature review to develop 15 search terms for adolescent SRH–related apps in the Canadian and US Apple and Google app stores. After generating the search results, inclusion and exclusion criteria were applied. Using the remaining apps, we built an evidence table of app information, and app reviewers assessed each included app using the Mobile App Rating Scale. App assessments were then used to highlight trends between apps and identify gaps in app quality.

**Results:**

In total, 2761 apps were identified by our searches, of which 1515 were duplicates. Of the 1246 remaining apps, 15 met the criteria for further assessment. Across all subdomains, on a scale of 1-5, the mean app score was 3.4/5. The Functionality subdomain had the highest mean score of 4.1/5, whereas the Engagement subdomain had the lowest score of 2.9/5. The top 4 apps were Tia: Female Health Advisor (4.7/5), Under the Stethoscope (4.2/5), Condom Credit Card (4.1/5), and Shnet (3.7/5).

**Conclusions:**

This environmental scan aimed to provide a comprehensive overview of the mobile apps developed to promote adolescent SRH knowledge and outcomes. Of the 15 mobile apps available to provide information related to adolescent SRH, few provided comprehensive, reliable, and evidence-based SRH information. Areas of strength included the apps’ gestural design, performance, ease of use, and navigation. Areas of weakness included app goals, evidence base, and app customization options. These results can be used to conduct future studies evaluating the use and efficacy of mobile apps on health knowledge and behaviors and promote adolescent SRH.

## Introduction

Adolescence is a critical period in the transition from childhood into adulthood, during which young individuals aged 10 to 19 years experience substantial physical, psychological, social, and emotional changes [1]. Adolescents are a vulnerable population because of their age-related psychosocial and biological changes and the challenges associated with navigating these changes [2]. As part of their physical, psychological, and social development, it is common for adolescents to explore their sexual identities and feelings [3]. Neglecting adolescents’ sexual and reproductive health (SRH) needs can affect their physical and mental health, future employment, economic well-being, and ability to reach their full potential [4-6].

Interventions to promote adolescent SRH (ASRH) increasingly use mobile phones. Mobile app platforms have the potential to advance SRH. Nearly 90% of young people aged 15 to 24 years in North America use the internet daily or own a smartphone [7,8]. The use of mobile technology for health promotion offers privacy [9-14], access to personalized information [9,11,13,15], and convenience [9,14,16], making it a valuable way to provide accurate information to adolescents about sexual health [9-15]. Furthermore, young people are responsive to and excited about using new technologies for SRH promotion [9,12,17,18]. Offering SRH information via mobile technologies has an emerging evidence base that recommends mobile health (mHealth) as an acceptable, feasible, and promising intervention approach [19-22]. This evidence includes, first, the World Health Organization–led High Impact Practices recommendation that digital technologies be integrated into family planning [19]. This recommendation is supported by a review of SMS text messaging as a digital tool [20] that demonstrates the high acceptability of these interventions among beneficiaries, even though few apps were available with this evaluative component [20]. Echoing these recommendations, a systematic review of mHealth added that the outcomes of these interventions were generally positive but susceptible to threats such as a lack of stable development funding [21]. Although a second systematic review also concluded that these interventions were promising, both reviews end by exhorting the collection of additional evidence [22]. Previous research suggests that providing SRH information via mobile phones is highly appealing to young people and can positively influence SRH outcomes including improving knowledge, reducing sexual risk behavior, and increasing the use of health services [23-27]. The appealing qualities of mHealth interventions (eg, mobile apps) have translated into growing recognition that mobile apps offer a promising platform for reaching large numbers of adolescents across diverse settings with private, essential, high-quality, and comprehensive SRH information and support.

Given the rapid proliferation of smartphone apps, there are several mobile apps that have been developed to promote ASRH. However, to date, no comprehensive attempt has been made to identify and provide information on the quality of these apps. It is increasingly difficult for users, health professionals, and researchers to readily identify and assess high-quality apps [28]. Little information on the quality of these apps is available, beyond the star ratings published on retailers’ web pages, whereas app reviews are subjective by nature. An updated systematic approach to establishing and evaluating the quality of ASRH mobile apps is urgently needed to rigorously evaluate whether they are a viable and effective strategy for reaching adolescents and improving ASRH knowledge and behaviors in particular. Although recent reviews have examined digital health solutions to ASRH [9,29-31], none have updated our knowledge by using the same evaluative framework to directly compare the quality of these solutions. The objective of our study was to conduct an environmental scan to produce an inventory of ASRH-specific mobile apps with descriptions of their purpose, structure, operations, and quality of evidence. An understanding of the available ASRH-specific mobile apps that currently exist in North America will help inform (1) the quality and usability of mobile apps to promote ASRH and (2) the potential development of new mobile apps specific to adolescents living in North America.

## Methods

### Summary

We developed search terms designed to work with Apple and Google’s app store search algorithms, and then, using software built for searching both stores, we created an app database based on this search strategy. Subsequently, 2 reviewers applied the inclusion and exclusion criteria to the database and filtered apps based on 9 criteria. Using this list of apps, we built an evidence table of app information, and the app reviewers assessed each included app using a validated health app assessment framework, the Mobile App Rating Scale (MARS) [32,33]. Discrepancies between ratings were addressed through discussion between the app reviewers. Results were then analyzed for trends in SRH-related apps for adolescents, and gaps in app quality that could be used to improve apps in the future were identified.

### Search Terms

As we were interested in SRH apps for adolescents in North America, we limited searches to the US and Canadian versions of the Google Play and Apple App stores. There is little formalized knowledge available to researchers about the specifics of how these stores’ searches work [34], and our results will be presented in the context that we lack specificity about how these algorithms work. Based on outreach to Google and Apple, as well as available documentation for app developers, results from the Google app store are drawn from app title, publisher, and app description, whereas results from the Apple app store are based on app title, keywords, and primary category (eg, education or lifestyle).

When searching Apple and Google’s app stores using a mobile device, we found that apps unavailable in Canada or the United States and apps not compatible with a particular device were not included in search results. In addition, results were personalized, which could have biased the apps examined based on the researchers’ search profiles. We addressed this by using custom software built to search the Google Play and Apple App stores that has been previously tested to ensure that personalized results and device compatibility were not influencing search results [35].

We carried out a short literature scan on ASRH using 4 electronic databases (Ovid MEDLINE, PubMed, Cochrane Library, and CINAHL) to identify 15 key terms related to ASRH: sex, sexuality, sexual health, sexual education, sexual health education, reproductive, reproductive health, contraceptive, birth control, pregnancy, safe sex, sex and relationships, sexually transmitted disease (STD), sexually transmitted Infection (STI), and HIV.

### Search and Screening

We searched the Apple and Google app stores in Canada and the United States on December 19, 2020, using the 15 terms above. The search returned a maximum of 50 results per search term per country, for each store, for a maximum of 1500 results per app store (750 apps each from the Canadian and US stores). We used custom Python software (Python Software Foundation) that is not susceptible to changes in results from search personalization and device limitations, as confirmed by testing on different computers. Results were automatically organized in a CSV database, which was exported to an Excel spreadsheet (Microsoft Corporation), and paired with an Excel-ready version of the MARS to allow it to be integrated easily into our evidence table.

In total, 6 inclusion criteria and 3 exclusion criteria were used to screen the apps (Textbox 1).

The 2 app reviewers (SLP and SK) independently assessed app titles and metadata (eg, description and paid/free status). Reviewers discussed apps where disagreement on inclusion occurred and used additional information (eg, photos of the app from the Google or Apple store) to reach a consensus. In addition, the 2 app reviewers recorded the reasons why any app was unusable, unavailable, or could otherwise not be assessed using the MARS.

**Inclusion and exclusion criteria.**

**Inclusion criteria**

- Contains content related to sexual health education
- Addressed any component of sexual health or sexuality
- App’s intended audience includes adolescents (aged 10-19 years)
- App still exists in the Google Play or Apple App store while being assessed
- Targeted to North American adolescents (app specifically mentions adolescents as users)
- Available in English

**Exclusion criteria**

- Paid (purchased; these apps, which only account for 5% of all apps [36], are unlikely to be useful to adolescents who cannot access, or are unwilling to access, a credit card)
- Developed for specific event such as a conference
- Targeted to a non–North American context

### App Quality Assessment

We used the MARS, a validated tool used for assessing health apps. The MARS was chosen for its high internal consistency and interrater reliability. The MARS contains items related to both the characteristics of an app (eg, rating and time since last update) and app quality assessment. This assessment is divided into 5 subscales: Engagement (eg, how interesting or fun the app is to use); Functionality (eg, how easy the app is to use); Aesthetics (eg, the visual appeal of the app); Information Quality (eg, information quality and relevance); and Subjective Quality (eg, how often the app would be used). Subscales are further divided into items (directed questions). All MARS items are scored on a 5-point, Likert-type scale, with a high score indicating favorability for that item.

The 2 reviewers (SLP and SK) trained on the MARS by reviewing previously rated apps, practiced rating 5 health apps as a group with all authors, and then discussed discrepancies in ratings before carrying out the MARS assessment for all included apps. Differences on item scores were compared and discussed, and a final item score was agreed on by both reviewers. All differences in item scores were resolved following discussion.

To assess each app, reviewers installed the app on an Android or Apple device. If an app was available on both devices, the Apple version was assessed. Once installed, reviewers launched each app and interacted with it for 10 minutes. Reviewers created a log-in or account for apps that required this process to access app content. After interacting with the app, reviewers assessed it with the MARS scale and reaccessed portions of the app ad hoc to determine item scores. To determine whether an app had an evidence base present in scientific literature, reviewers searched Google Scholar and PubMed using the app’s name, and the first 50 results were examined for relevance. If a matching paper was found, reviewers examined the nature of that study to determine how to score the MARS item.

### Analysis

Once we built an evidence table for all included apps, we calculated subscale scores for each app by averaging items within each subscale and then calculated the MARS score by averaging the MARS items for that app. We converted subscale and MARS scores to a score out of 5 to match the MARS item score range. The mean score of each item was also calculated to create an item-wise mean score. We rank-ordered apps by MARS score to allow for comparisons of app quality.

We then analyzed apps by comparing scores between each app’s MARS score, subscale scores, and item scores. The subscale and item scores of the top 4 apps (with the number of apps arbitrarily chosen post hoc) were compared to the mean app score. Apps were also compared based on duration since the last update (dividing apps into less than or more than 6 months since the last update). To identify gaps in app quality, we compared mean item scores. We also identified areas for quality improvement in the top 4 apps by comparing each app’s item scores to the mean app item scores.

## Results

### Study Characteristics

**Figure 1:**
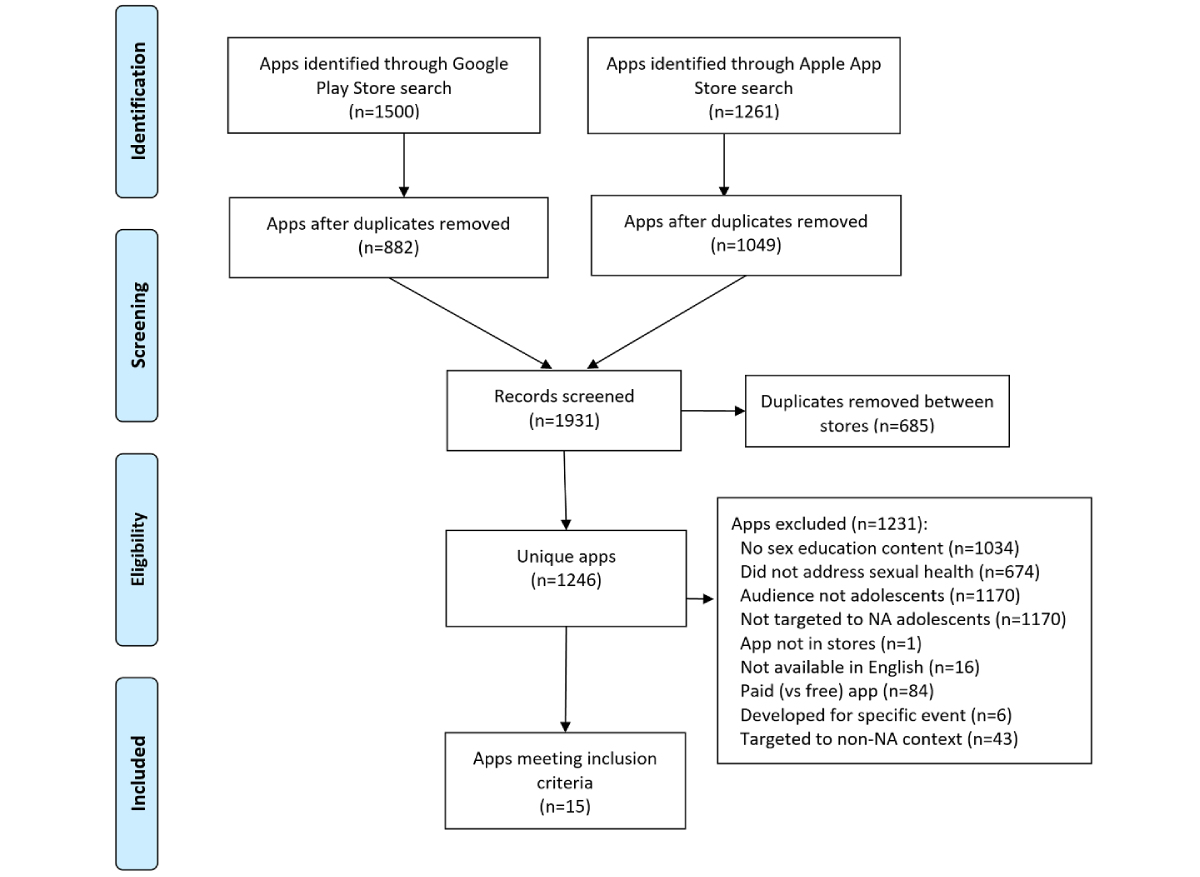
PRISMA (Preferred Reporting Items for Systematic Reviews and Meta-Analyses) diagram of the app search and screening process. NA: North America.

The search strategy (summarized in Figure 1) [37] identified a total of 2761 mobile apps across 4 searches. After removing 1515 duplicate apps from the results, a total of 1246 mobile apps from the Apple App store (n=751) and Google Play store (n=495) were screened against the inclusion and exclusion criteria. Based on the final inclusion criteria, 15 mobile apps were included in our environment scan. Of the 15 mobile apps included, 5 were only available from the Apple platform (Condom Credit Card; Teenagers with Experience; Tia: Female Health Advisor; TMI Georgia; and Under the Stethoscope), 3 were only available from the Google platform (Adolescent Health Issues; Class 12 Bio Notes; and Sexual Reproductive Health Counsellor), and 7 were available from both Apple and Google platforms (bMOREsafe; It Matters; My Sex Doctor Lite; NeedTayKnow; Shnet; SAUTIplus; and The Sex Talk). Of the 15 mobile apps included, 4 were updated within the last 6 months at the time of analysis (bMOREsafe; NeedTayKnow; Shnet; and Tia: Female Health Advisor), whereas 8 were last updated more than 6 months ago (Adolescent Health Issues; Class 12 Bio Notes; Condom Credit Card; It Matters; My Sex Doctor Lite; SAUTIplus; Sexual Reproductive Health Counsellor; and TMI Georgia). The remaining 3 mobile apps did not provide information on the date of the last update (Teenagers with Experience; The Sex Talk; and Under the Stethoscope). Additionally, 4 mobile apps were targeted specifically for adolescents (Adolescent Health Issues; Class 12 Bio Notes; Teenagers with Experience; and Under the Stethoscope), 8 were targeted for adolescents and young adults (Condom Credit Card; It Matters; NeedTayKnow; SAUTIplus; Sexual Reproductive Health Counsellor; Shnet; The Sex Talk; and TMI Georgia), and 3 were targeted for adolescents, young adults, and adults (bMOREsafe; My Sex Doctor Lite; and Tia: Female Health Advisor). This information is summarized in Table 1.

**Table 1:** Assessed app characteristics.

App name	Availability				Time since update				Target(s)		
	Apple	Google	Both	<6 months		>6 months	No info	Adolescents		Adolescents and young adults	Adolescents, young adults, and adults
Sexual Reproductive Health Counsellor		✓				✓				✓	
Adolescent Health Issues		✓				✓		✓			
Class 12 Bio Notes		✓				✓		✓			
My Sex Doctor Lite			✓			✓					✓
NeedTayKnow			✓	✓						✓	
SAUTIplus			✓			✓				✓	
bMOREsafe			✓	✓							✓
Teenagers with Experience	✓						✓	✓			
The Sex Talk			✓				✓			✓	
It Matters			✓			✓				✓	
TMI Georgia	✓					✓				✓	
Shnet			✓	✓						✓	
Condom Credit Card	✓					✓				✓	
Under the Stethoscope	✓						✓	✓			
Tia: Female Health Advisor	✓			✓							✓

### Study Findings

The included mobile apps had a mean score of 3.4/5 on the MARS. The included mobile apps scored the highest on the subdomain of Functionality, with a mean score of 4.1/5, whereas the subdomain of Engagement received the lowest mean score of 2.9/5. The overall mobile app mean score was 2.5/5 for the Subjective Quality subdomain, 3.3/5 for the Aesthetics subdomain, and 3.3/5 for the Information subdomain. The scores are summarized in Table 2.

**Table 2:** Scores by Mobile App Rating Scale (MARS) subdomain and overall 1uality.

			Mean score^a^
**Subdomain**			
	A: Engagement	2.9	
	B: Functionality	4.1	
	C: Aesthetics	3.3	
	D: Information	3.3	
	E: Subjective Quality	2.5	
**App quality**			
	Overall mean (subdomains A to D)	3.4	

^a^Mean scores are out of a total of 5.

### MARS Subdomains

**Figure 2:**
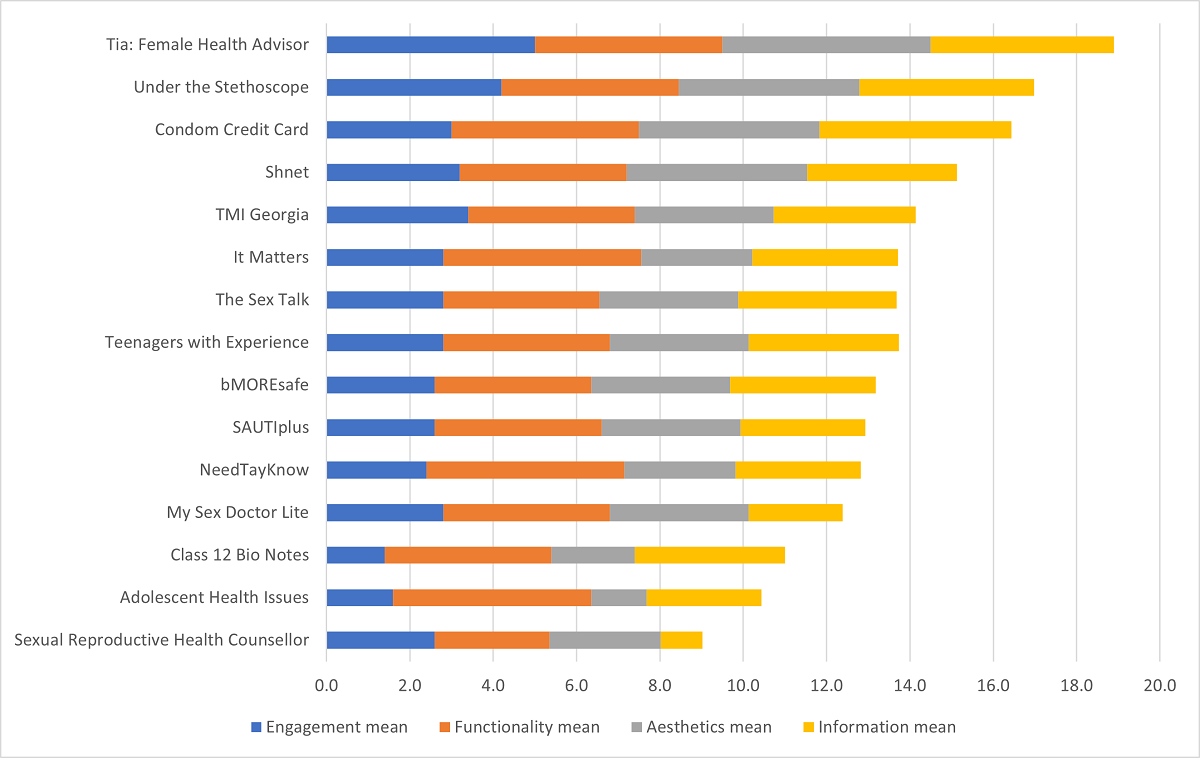
Cumulative score of each app’s Mobile App Rating Scale (MARS) subdomains.

The individual mobile app MARS scores varied between 2.3/5 and 4.7/5 on the MARS tool (Figure 2). Tia: Female Health Advisor was the highest-scoring mobile app, followed by Under the Stethoscope, Condom Credit Card, and Shnet.

For the Engagement subdomain, Tia: Female Health Advisor received the highest mean score of 5/5, whereas Under the Stethoscope received a mean score of 4.2/5. Shnet and Condom Credit Card had lower mean scores of 3.2/5 and 3/5, respectively. For the Functionality subdomain, Tia: Female Health Advisor and Condom Credit Card both received the highest mean score of 4.5/5. Under The Stethoscope followed with a mean score of 4.3/5, whereas Shnet received a Functionality mean score of 4/5. In the Aesthetics subdomain, Tia: Female Health Advisor again received the highest mean score of 5/5. Under the Stethoscope, Condom Credit Card, and Shnet each received a mean score of 4.3/5 in the Aesthetics subdomain. The mean scores in the Information subdomain were 4.6/5 for Condom Credit Card, 4.4/5 for Tia: Female Health Advisor, 4.2/5 for Under the Stethoscope, and lastly, 3.6/5 for Shnet. For the Subjective Quality mean scores, Tia: Female Health Advisor was rated the highest with a mean score of 4.5/5. Under the Stethoscope and Shnet both received a Subjective Quality mean score of 3.8/5, whereas Condom Credit Card received the lowest Subjective Quality mean score of 3.5/5.

### Areas of Strength

**Figure 3:**
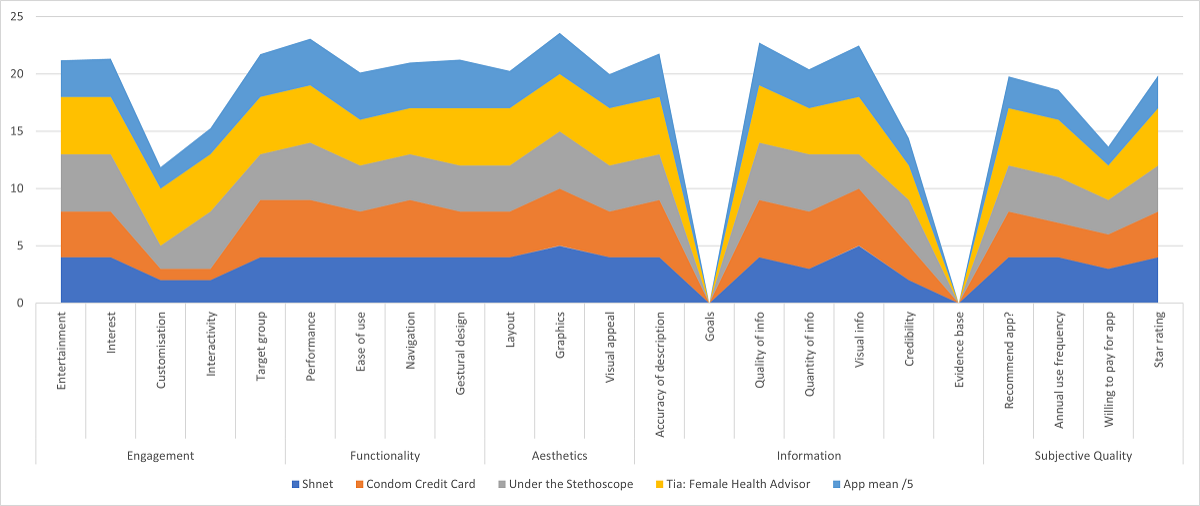
Scores of top 4 apps and the mean of all apps by Mobile App Rating Scale (MARS) items.

In a comparison of the top 4 mobile apps against the mean score of all included mobile apps (Figure 3), our study found common areas of strength on the MARS items. Overall, the mobile apps included in our study scored high on the MARS items of *gestural design*, *performance*, *ease of use*, and *navigation* (Figure 3). Although *visual information* was rated highly (4.5/5), only 8 (53%) out of 15 apps contained it, and therefore, this item was not considered a strength. These results suggest that future mobile apps in the area of SRH should continue to consider gestural design, performance, ease of use, and navigation in the development and dissemination of mobile apps. However, our study identified important gaps in the MARS items for current mobile apps being offered in the area of SRH.

### Areas of Weakness

#### Primary Areas of Weakness

In a comparison of the top 4 mobile apps against the mean score of all included mobile apps (Figure 3), our study identified primary and secondary areas of weakness on the MARS items for mobile apps currently being offered.

Our study identified 2 major gaps in the MARS items across the included mobile apps. First, the MARS item *goals*—“Does app have specific, measurable and achievable goals (specified in app store description or within the app itself)?” [32]—was absent across mobile apps. Second, the MARS item *evidence base*—“Has the app been trialled/tested; must be verified by evidence (in published scientific literature)?” [32]—was additionally lacking across mobile apps. These findings suggest that researchers should strongly consider incorporating specific goals in the development of SRH mobile apps, in addition to developing a strong evidence base for future mobile apps on SRH. Integrating these MARS items—*goals* and *evidence base*—into future SRH mobile apps may fill the current gap in end-user needs.

#### Secondary Areas of Weakness

Furthermore, our study identified 2 secondary areas of weakness in a comparison of the top 4 mobile apps against the mean score of all included mobile apps. First, the MARS item *customisation—*“Does it provide/retain all necessary settings/preferences for apps features (e.g. sound, content, notifications, etc.)?” [32]—was lacking. Future development of mobile apps on SRH topics should consider the importance of app customization to comprehensively reach the needs of potential end users. The MARS item *Would you pay for this app?* additionally scored low across the top 4 mobile apps assessed. By addressing the aforementioned gaps of *goals*, *evidence base*, and *customisation*, future SRH mobile apps may increase end-user satisfaction, and thus, increase end users’ willingness to pay for the app.

## Discussion

### Principal Findings

This environmental scan aimed to provide a comprehensive overview of the mobile apps developed to promote ASRH knowledge and outcomes. Research into smartphone apps for ASRH is sparse. Despite the plethora of mobile apps on the market, our literature search identified only 15 apps pertaining to this subject. To our knowledge, our study is one of the first to perform a comprehensive assessment of mobile apps being developed for ASRH that are used during the COVID-19 pandemic. Smartphone apps have immense potential to improve health knowledge, behaviors, and outcomes for young people. We identified 15 mobile apps that we scored across the MARS. These apps differed in terms of their engagement, functionality, aesthetics, information quality, and overall purpose. Most of the apps acquired the highest score in Functionality (mean score of 4.1). This finding shows that most apps prioritize functionality (including concepts such as app performance, the ease of learning the app, and easy navigation between screens) over other features.

The majority of the mobile apps included in this environmental scan are lacking in the MARS items *goals* and *evidence base*. This finding is unsurprising as the mHealth field has been criticized for producing limited evidence about the efficacy and effectiveness of evidence base information [38-40]. Researchers of previous studies that examined youths’ perspectives on the use of digital technologies in sexual health education reported that adolescents prefer sexual health education resources that are accessible (ie, a mobile app is a preferred resource because they receive immediate answers to their questions), trustworthy (ie, resources must be credible and have an evidence base), and confidential and private (ie, resources should offer information in a nonthreatening way that will not cause embarrassment) [41,42]. The credibility, quality, and accuracy of information are important factors that encourage young people to use digital platforms for SRH information, and adolescents do not act on digital information if they do not trust its credibility [9,43]. Digital technologies or social media platforms with improved resources that provide evidence-based information on SRH and rights are useful for accessing reliable and confidential information [44], but the availability of this information in some domains (eg, HIV-related apps) has been criticized [45].

Another area of weakness identified is the lack of app customization to comprehensively reach the needs of all potential end users. Consultation with users is essential in the development of mobile apps targeted at young people, as this group can be particularly influenced by the look and feel of an app. Previous research suggests that listening to and meeting young people’s desires in terms of mobile app and content is essential in engaging them [46-48].

### Strengths and Limitations

The psychometric properties of the MARS tool have been proven to be reliable and valid [32], and the use of this tool lends strength to our study’s conclusions. Further, our study provides a comprehensive assessment of all mobile apps available in North America for adolescents’ SRH. However, apps in languages other than English could not be assessed, which limits the generalizability of our results and the stores in which we could search for apps. Likewise, paid apps were not included in the search. In addition, the features of the apps examined by us may be different from the updated versions of the app, and these features might have been addressed in apps developed after this review. This possibility is inevitable considering the rapidity with which apps are developed and reformed. Despite the high interrater reliability of this scale [32], the reviewers’ subjectivity might have influenced the ratings awarded, and caution must be exercised when interpreting the results portrayed in this study. Finally, only 2 reviewers carried out the MARS assessment of each app, limiting information quality in our analysis.

### Conclusions

Digital health tools, such as smartphone-based apps, play an important role in preserving the continuity of SRH services for adolescents and youths. There are numerous mobile apps available to provide information related to ASRH. However, very few mobile apps provide comprehensive, reliable, and evidence-based SRH information to promote ASRH. This review provides an overview of mobile apps available in North America related to ASRH, summarizes their strengths and limitations through a qualitative assessment, and delineates key functions and features needed for future apps. This information can be used to conduct future studies to evaluate the use and efficacy of mobile apps on health knowledge and behaviors and promote ASRH.

## Abbreviations

ASRH: adolescent sexual and reproductive health
MARS: Mobile App Rating Scale
mHealth: mobile health
SRH: sexual and reproductive health
